# Comparisons of Native Shiga Toxins (Stxs) Type 1 and 2 with Chimeric Toxins Indicate that the Source of the Binding Subunit Dictates Degree of Toxicity

**DOI:** 10.1371/journal.pone.0093463

**Published:** 2014-03-26

**Authors:** Lisa M. Russo, Angela R. Melton-Celsa, Michael J. Smith, Alison D. O'Brien

**Affiliations:** Department of Microbiology and Immunology, Uniformed Services University of the Health Sciences, Bethesda, Maryland, United States of America; Wadsworth Center, New York State Dept. Health, United States of America

## Abstract

Shiga toxin (Stx)-producing *E. coli* (STEC) cause food-borne outbreaks of hemorrhagic colitis. The main virulence factor expressed by STEC, Stx, is an AB_5_ toxin that has two antigenically distinct forms, Stx1a and Stx2a. Although Stx1a and Stx2a bind to the same receptor, globotriaosylceramide (Gb3), Stx2a is more potent than Stx1a in mice, whereas Stx1a is more cytotoxic than Stx2a in cell culture. In this study, we used chimeric toxins to ask what the relative contribution of individual Stx subunits is to the differential toxicity of Stx1a and Stx2a *in vitro* and *in vivo*. Chimeric *stx_1_/stx_2_* operons were generated by PCR such that the coding regions for the A_2_ and B subunits of one toxin were combined with the coding region for the A_1_ subunit of the heterologous toxin. The toxicities of purified Stx1a, Stx2a, and the chimeric Stxs were determined on Vero and HCT-8 cell lines, while polarized HCT-8 cell monolayers grown on permeable supports were used to follow toxin translocation. In all *in vitro* assays, the activity of the chimeric toxin correlated with that of the parental toxin from which the B subunit originated. The origin of the native B subunit also dictated the 50% lethal dose of toxin after intraperitoneal intoxication of mice; however, the chimeric Stxs exhibited reduced oral toxicity and pH stability compared to Stx1a and Stx2a. Taken together, these data support the hypothesis that the differential toxicity of the chimeric toxins for cells and mice is determined by the origin of the B subunit.

## Introduction

Shiga toxin (Stx)-producing *E. coli* (STEC) are Gram-negative, enteric pathogens with an estimated infectious dose of less than 50 organisms [1]. Among the multiple serotypes associated with disease, O157:H7 is responsible for more than 63,000 of the 175,000 total estimated STEC cases each year [2]. Ruminants, especially cattle, are the natural carriers of STEC, and these bacteria most commonly enter the food chain during beef processing [3]–[6]. Outbreaks and sporadic cases of STEC infection have also been attributed to contaminated fresh produce, person-to-person spread, and environmental sources [6], [7]. Upon *E. coli* O157:H7 STEC infection of humans, the most common disease manifestation is hemorrhagic colitis. A more severe sequela, the hemolytic uremic syndrome (HUS), characterized by microangiopathic hemolytic anemia, thrombocytopenia and acute kidney failure, occurs in 5–15% of *E. coli* O157:H7-infected individuals [8]–[11].

Stx is the primary virulence factor associated with disease caused by STEC. This group of organisms may encode for Stx1a and/or Stx2a, two biologically similar, though antigenically distinct toxins with analogous crystal structures and identical modes of action [reviewed in [12]]. The Stxs are AB_5_ toxins; the A subunit, which has a protease sensitive site near the C-terminus, is cleaved into two parts. The A_1_ subunit is responsible for the catalytic activity of the toxin molecule, and the A_2_ peptide, which threads through the center of the B pentamer, links the binding moiety to the catalytic subunit [13], [14]. The homopentameric B subunit binds to the host cell receptor, globotriaosylceramide (Gb3) [15], a glycolipid that that is primarily expressed on endothelial cells. After Stx binds Gb3, the toxin undergoes retrograde transport through the Golgi network to the endoplasmic reticulum [16]. The A_1_ subunit is released into the cytoplasm from the endoplasmic reticulum and depurinates a single adenine residue from the 28 s RNA of the 60 S ribosome, an injury that halts protein synthesis and leads to cell death [17], [18].

Although Stx1a and Stx2a are biologically similar, Stx2a is associated with an increased number of outbreaks and more severe disease [19]–[24]. These latter observations correlate with reports that Stx2a has a parenteral 50% lethal dose (LD_50_) for mice that is 100-fold lower than is Stx1a [25], [26]. Conversely, Stx1a has a 50% cytototoxic dose (CD_50_) for Vero cells that is 10 times lower than is Stx2a [26]. Multiple methods and approaches have been used to study the paradox of the differential *in vitro* (on Vero cells) and *in vivo* (in mice) toxicities of Stx1a and Stx2a. There is no difference in the enzymatic activity between Stx1a and Stx2a in a cell-free rabbit reticulocyte lysate assay of protein synthesis inhibition [26], [27]; therefore, the A subunit is not responsible for the differential toxicity of Stx1a and Stx2a, at least at the level of the ribosome. Multiple studies propose that the biological differences between Stx1a and Stx2a are specific to the B-subunit. For example, Stx1a binds Gb3 *in vitro* with a greater affinity than does Stx2a [25], [26], [28], [29], a finding that may explain the greater toxicity of Stx1a on Vero cells. In contrast, *in vivo*, a higher binding affinity for the receptor may reduce overall toxicity if secondary targets (non-lethal) are bound. Indeed, after intravenous (iv) injection into mice radiolabelled Stx1a demonstrates increased binding to murine pulmonary and splenic tissues but decreased kidney binding (kidney damage is responsible for the lethality of the Stxs to mice in our hands [30], [31]), and a shorter serum half-life, compared to Stx2a [32].

Chimeric Stxs have been used to study the contribution of the individual A and B subunits to toxin function. Ito *et al*. purified individual toxin subunits and then recombined them to form recombinant Stx2a, and chimeras Stx1aA/Stx2aB2 and Stx2aA/Stx1aB [33]. Although that group observed equivalent cytotoxicity for the recombinant Stx2a as compared to native Stx2a and found no difference in the cytotoxicities of Stx1aA/Stx2aB2 and Stx2aA/Stx1aB, they did not report Stx1a activity. Because of the lack of data for Stx1a and the relative equivalent toxicity of Stx1aA/Stx2aB2 and Stx2aA/Stx1aB, no conclusion about the relative contribution of the A or B subunits to toxicity of the prototype toxins could be made [33]. Another study took a similar recombinant approach to produce and then assess chimeric Stx function, and showed that the cytotoxicity of the chimerics correlated with the source of the B subunit; however, the activity of all toxins tested was below what would be expected, a result that may indicate the recombined toxins did not fold properly [27]. Weinstein *et al*. tried two genetic approaches (operon fusions and co-transformation of two compatible plasmids that each encoded an individual toxin subunit into a K-12 strain) to produce hybrid Stx1a/Stx2a molecules but neither method produced a functional Stx1aA/Stx2aB2 chimeric [34]. Furthermore, the Stx2aA/Stx1aB chimeric had a cytotoxic profile that was closer to that of Stx2a than Stx1a, but the caveat to that result is that the level of toxin in the preparations was not quantitated [34]. Another group that used an operon fusion to produce an Stx2aA/Stx1aB chimera reported that that hybrid toxin had cytotoxicity, Gb3 binding, and LD_50_ values that were intermediate between the Stx1a and Stx2a values [35]. Taken together these studies do not provide a definitive answer to the question of which toxin subunit is responsible for the differential toxicity of Stx1a and Stx2a for Vero cells and mice.

We believe that previous analyses of hybrid Stxs may have been limited by decreased stability of the chimeric toxins. Because the A_2_ peptide is critical for native holotoxin stability [36], our approach was to create chimeric Stxs in which the A_2_ peptide comes from the same origin as the B subunit. We then assessed those toxins for purity, cytotoxicity, and lethality in mice by the intraperitoneal (ip) and oral routes. We found that the toxicity of the chimeric toxins for Vero and HCT-8 cells, and by ip delivery into mice, correlated with the origin of the B subunit. However, the chimeric Stxs exhibited reduced activity after oral intoxication.

## Material and Methods

### Ethics Statement

All animal studies were approved by the Institutional Animal Care and Use Committee of the Uniformed Services University of the Health Sciences. These studies were conducted in strict accordance with the recommendations of the Guide for the Care and Use of Laboratory Animals [37]. Animals were housed in an environmentally controlled room approved by the American Association for Accreditation of Laboratory Animal Care (AAALAC).

### Bacterial strains and growth conditions

*E. coli* K12 DH5α strains were transformed to encode the native Stxs (Stx1a, pLPSH3 [38]; Stx2a, pJES120 [30]) or the chimeric Stxs, as described below. All bacterial strains were grown in Luria Bertani (LB) broth or LB agar supplemented with 100 μg/mL ampicillin for maintenance of all recombinant plasmids.

### Construction of chimeric toxin operons

Chimeric s*tx_1_A_1_: stx_2_A_2_ stx_2_B* and s*tx_2_A_1_: stx_1_A_2_ stx_1_B* operons were created by splice by overlap extension (SOE) PCR as previously described [39]. Specifically, the Stx1a clone, pMJS1, was used to amplify the PCR products s*tx_1_A_1_* and *stx_1_A_2_ stx_1_B* using primers MJS1 [39] and 2A_2_/1A_1_ (5′CTCTCTTCATTCACGGCGCGAACAGATCGCGATGCATGATGATGACAATTCAG-3′) and primers 1A_2_ ( 5′GTTGCCAGAATGGCATCTGATGAG-3′ ) and MJS2, respectively [39]. The Stx2a clone, pMJS2, was used to amplify the PCR products *stx_2_A_1_* and *stx_2_A_2_ stx_2_B* with primers MJS5 [39] and 1A_2_/2A_1_ (5′CTCATCAGATGCCATTCTGGCAACACGCGCCCCCTGATGATGGCAATTCAG-3′) primers MJS6 [39] and 2A_2_ (5′TCTGTTCGCGCCGTGAATGAAGAGAG-3′), respectively. The s*tx_1_A_1_* and *stx_2_A_2_ stx_2_B* amplification products were spliced together by PCR to create the 122 operon (with the junction from Stx1a to Stx2a after first R of RSVR in Stx1a). The *stx_2_A_1_* and *stx_1_A_2_ stx_1_B* amplification products were spliced together by PCR to create the 211 operon (with the junction from Stx2a to Stx1a after first R of RVAR in Stx2a). The PCR products were ligated into pBluescript II KS(-) (Stratagene, La Jolla, CA), transformed into *E. coli* DH5α, and named pMJS122 and pMJS211, respectively.

### Purification of Stx1a, Stx2a, and chimeric toxins

All four toxins (Stx1a, Stx2a, 211 and 122) were purified by affinity chromatography with five mL AminoLink Coupling Resin (Thermo Scientific) columns, as described previously [40]. Briefly, monoclonal antibody to either the Stx1a or Stx2a B subunit was covalently bound to the column resin in pH 7.2 coupling buffer according to manufacturer's instructions. Monoclonal 13C4 [41], purified from hybridoma supernatant, was used for purification of Stx1a and chimeric 211 (approximately 7 mg 13C4/column) while monoclonal BC5 BB12 [42], from ascites fluid, a gift from Dr. Nancy Strockbine, was used for Stx2a and chimeric 122 purification (approximately 13 mg/column). An independent column was used for the purification of each toxin.

#### Toxin purification

The *E. coli* K12 strain that contained the plasmid that encoded for the *stx* of interest was grown overnight, sedimented by centrifugation (5,000×g), resuspended 40X in sonication buffer (50 mM NaPO_4_, 200 mM NaCl), and disrupted by sonication. The cell lysate was sedimented by centrifugation (20,000×g) and placed over the appropriate affinity column. After purification, Stx was dialyzed against PBS in Slide-A-Lyzer dialysis cassettes (Thermo Scientific) and concentrated (Millipore Amicon Ultra 30 K) according to manufacturer's instruction, if necessary. The protein concentrations of the Stx preparations were determined with a bicinchoninic acid (BCA) assay (Thermo Scientific).

### Specific toxin concentration determination

Differences in the purity of toxin preparations were normalized as described previously [40]. Briefly, purified toxins were separated on a sodium dodecyl sulfate-polyacrylamide gel (SDS-Page), and the gel was stained with Oriole fluorescent stain (BioRad). The stained gel was scanned with ImageQuant LAS 4000 (GE Healthcare) and analyzed with ImageQuant TL software (GE Healthcare). The A and B subunits of each toxin bound the Oriole fluorescent stain similarly (Figure S1). Densitometry analysis of the Oriole-stained gel was used to determine the percent of the total protein bands that comprised the A and B subunit bands. Then the percentage of the preparation that could be attributed to toxin was multiplied by the total protein in each sample (as determined by BCA assay). Densitometry analysis of the A and B subunit bands also confirmed the expected 1∶5 subunit ratio, respectively, in each preparation.

### Cell culture

Vero cells (CCL-81, American Type Culture Collection [ATCC], Manassas, VA) were maintained in Eagle's minimal essential medium (EMEM) (Lonza, Inc., Walkersville, MD) while HCT-8 cells (CCL-244, ATCC), a human epithelial colorectal adenocarcinoma cell line, were maintained in RPMI-1640 medium (ATCC). All media were supplemented with 10% FBS, 10 U/mL penicillin, and 10 μg/mL streptomycin.

### Cytotoxicity assay

The Vero cytotoxicity assay has been described previously [43], [44]. Briefly, Vero cells were seeded in 96-well plates at a concentration of 10,000 cells per well and incubated at 37°C, 5% CO_2_ for 24 hours. Serial dilutions of toxin samples in fresh media (EMEM plus supplements as described above) were overlaid onto the cells, and the plates were incubated for an additional 48 h. The cells were then fixed with formalin and stained with crystal violet. The absorbance of the wells at 630 nm was measured spectrophotometrically. Cytotoxicity assays were done on HCT-8 cells in the same manner as for Vero cells, except that the cells were incubated for 48 hours prior to the application of toxin. For each toxin sample, CD_50_ was determined by the reciprocal of the toxin dilution that caused death of 50% of the cells in the monolayer compared to control cells. Specific toxin activity was calculated as the CD_50_/mL divided by the toxin concentration in mg/mL.

### Toxin-Gb3 binding enzyme-linked immunosorbent assay (ELISA)

An ELISA to quantitate Gb3-toxin binding was conducted as previously described [28]. Briefly, wells of an Immobilon 2HB 96-well plate (Thermo Electron Corp) were coated with decreasing concentrations of purified Gb3 glycosphingolipids (Matreya) suspended in 100% ethanol and dried overnight. The wells were then overlaid with 20 ng of toxin. Polyclonal rabbit anti-Stx1a and -anti-Stx2a antibodies (each at 1∶5000) were used in combination as the primary antibody for the detection of all four toxins, while goat anti-rabbit-HRP antibody (1∶2,000) was used as the secondary. Statistical significances of the differences in how well toxins bound to Gb3 were determined viaTwo-Way ANOVA with Tukey's adjustment for multiple comparisons.

### Transwell assay

We followed the protocol published by Hurley *et al*. to establish polarized HCT-8 cells [45]. Transwell permeable supports (Corning) with polycarbonate membrane transwell inserts were seeded with 2–5×10^5^ HCT-8 cells. Media was changed every two days until the cells were polarized (8–10 days) as determined by Millicell-ERS resistance reader (Millipore Corporation, Bedford, Mass.) and a transepithelial electrical resistance (TEER) above 2,000 Ω/cm^2^. Once the cells were polarized, 20 ng of toxin was added to the upper chamber. All cells remained polarized through 24 hours post-intoxication as the TEER did not drop below 2,000 Ω/cm^2^. The amount of toxin translocated from the apical to the basolateral side of the cells was determined as follows. A 40 μL sample of media from the lower chamber was removed and the toxicity in that sample measured on Vero cells. The Vero cell toxicity of the basolateral sample was compared to a standard toxicity curve of known toxin concentrations, and then the value was corrected for the total volume in the lower chamber. The statistical significance of the amount of translocated Stx was determined via the Students t-Test.

### Mice

All experiments were conducted with 5–6 week old, female BALB/c mice from Charles River Laboratories (Wilmington, MA). Mice were housed in filter-top cages with access to food and water ad libitum, unless otherwise stated.

### Lethal dose 50% (LD_50_) studies

Mice were injected by the ip route with 0.1 mL of toxin/PBS at varying dilutions for ip LD_50_ studies. The ip LD_50_ values were confirmed for the native toxins with a total of 25 or 41 mice for Stx1a and Stx2a, respectively (4–5 mice per dose). A total of 45 mice were used for the chimeric 122 studies, with 3–5 mice per dose. A total of 48 mice were used for the chimeric 211 ip LD50 studies, with 5–6 mice per dose. Mice were gavaged with 0.2 or 0.3 mL of toxin/PBS dilutions for the ig LD_50_ studies. Food and water were removed for 18 and 2 hours, respectively, prior to oral intoxication. A total of 20 mice were used for the chimeric 122 studies, while five mice were used for the chimeric 211 study. We used the minimum number of animals required to attain statistical significance. No analgesics were administered since non-steroidal anti-inflammatory drugs (NSAIDs) could confound or mask the effect of Stx, and alter the LD_50_ values. All mice were weighed daily and monitored at least twice per day for morbidity and mortality for two weeks. The animals were checked every 6 h during the time period when mortality was expected. Mortality was an endpoint for the LD_50_ studies, however mice that exhibited signs of extreme morbidity were humanely euthanized by an overdose of inhalational isoflurane followed by cervical dislocation or CO_2_ overdose, in accordance with the AVMA Guidelines on Euthanasia. Extreme morbidity was defined as two or more of the following symptoms: ≥25% weight loss, ruffled fur, lethargy, labored breathing, hunched posture, inability to remain upright, or impaired ambulation that prevents the animal from reaching food and water. LD_50_ and 95% confidence interval (CI) values were determined by Probit regression analysis with log transformation of the values. The relative median potency, defined as the ratio of two LD_50_s, along with the CI for the ratio, was calculated to determine statistical significance among the Stxs. If the calculated CI did not include 1.0, then the two LD_50_s were significantly different with P<0.05.

### Active toxin in feces of ig-intoxicated mice

Fecal samples were collected 3, 9, 12, 24, and 48 hours after ig intoxication (same mice from the ig LD_50_ studies). At each time point, mice were transferred to an individual cage with no bedding for 30–40 minutes. Fecal pellets were collected, weighed, and EMEM 1∶10 w/v was added. The fecal samples were frozen at −20°C until all time points were completed so that they could be analyzed simultaneously. The samples were thawed, mixed by vortex and then filter-sterilized (0.45 micron). The sterile fecal supernatant was applied to Vero cells as described for the cytotoxicity assay above, and the total level of toxicity present in each sample was determined. The basal level of fecal cytotoxicity was determined from stools of unintoxicated control mice, and that value was subtracted from all experimental sample values. The geometric mean for each group was then calculated. The amount of toxin present was then calculated by dividing the geometric mean CD_50_ by the specific activity of that toxin (CD_50_/ug). The Students t-Test was performed on log_10_ transformed cytotoxicity CD_50_ values.

### pH stability of Stxs

The effect of pH and temperature on toxin stability was measured as previously published [26]. The pH buffers used were: pH 7, 100 mM Tris; pH 5, 100 mM sodium acetate; and pH 3, 100 mM glycine. Briefly, toxin was diluted to 10^7^ CD_50_ in 0.2 mL of PBS. Twenty microliters of each toxin was then added to 180 μL of the pH buffers or PBS as a control. Samples were incubated at either 37°C or 60°C for one hour, then immediately placed on ice and neutralized 1∶10 v/v with 1 M Tris, pH 7.5. Toxin activity was then determined by the Vero cell cytotoxicity assay described above. To determine the fold change in Stx activity, the log transformed CD_50_ values from experimental samples were divided by the log transformed CD_50_ value of the PBS control. The mean value from four biological replicates is reported. Statistical significance of fold-change differences was determined with a 1-way ANOVA with Tukey's correction for multiple comparisons.

## Results

### Construction, purification, and analysis of chimeric and native toxins

**Figure 1:**
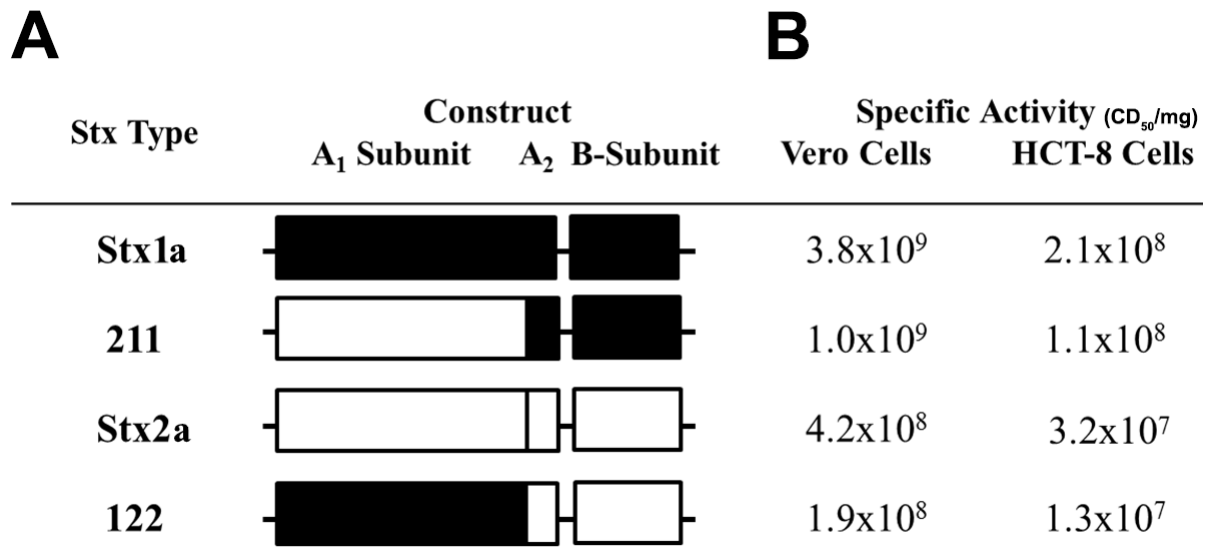
Native and chimeric Stx operon structure and activities. (A) Illustration that depicts the origin of the individual subunits, A_1_, A_2_, or B, in the native and chimeric operons. Stx1a is in black, while Stx2a is in white. The chimerics are named such that the number represents the native toxin from which that subunit originated. (B) Specific activities of the toxin panel after intoxication of Vero and HCT-8 cells. Geometric mean of representative of seven biological replicates.

We created two chimeric *stx_1_*/*stx_2_* operons, purified the toxins produced by those operons, and evaluated them in comparison with Stx1a and Stx2a in several assays as described below. The chimeric toxins consisted of the A_1_ subunit from either Stx1a or Stx2a and the A_2_ peptide and B subunit from the heterologous toxin (see schematic in Figure 1A). The chimeric toxins were named according to the origin of the A_1_, A_2_, and B subunits, i.e. 122 or 211. We determined the cytotoxicity profile of the toxin panel (Stx1a, Stx2a, 122, and 211) on Vero and HCT-8 cells (Figure 1B). The Vero cell specific activities of the native toxins were similar to those previously reported [26]; Stx1a was 3.8×10^9^ CD_50_/mg, while Stx2 was approximately 10-fold lower at 4.2×10^8^ CD_50_/mg (Figure 1B). The activity of the chimeric toxins was equivalent to the native toxin with the homologous B-subunit, such that 211 and 122 had specific activities of 1.0×10^9^ and 1.9×10^8^ CD_50_/mg, respectively. Similar toxicity results were obtained on HCT-8 cells, although the overall activity of the toxins was 10-fold lower than observed for Vero cells as expected (Figure 1B). (HCT-8 cells exhibit reduced sensitivity to Stx due to decreased expression of the toxin receptor, Gb3, on the cell surface [28]).

**Figure 2:**
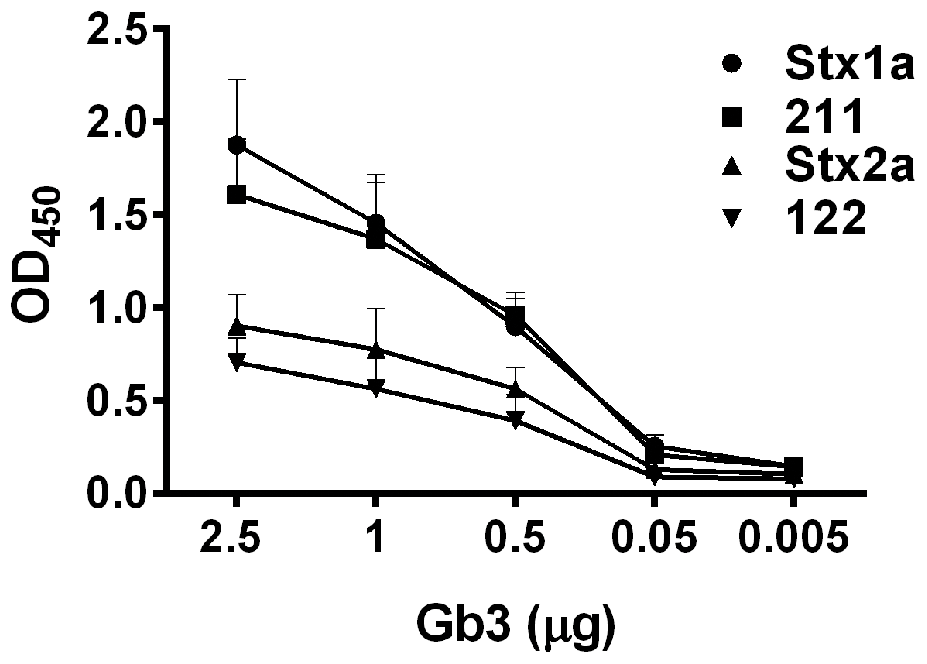
Binding of toxin panel to Gb3 as measured by ELISA. All four toxins bound Gb3; however, Stx1a and 211 bound Gb3 with a significantly higher affinity than Stx2a or 122 (p<0.01). Additionally, when toxins with identical B subunits were compared, Stx1a vs 211 and Stx2a vs 122, there was no difference in binding (p>0.05). Error bars represent standard error of the mean (SEM); n = four biological replicates. Toxin-Gb3 binding was compared by Two-Way ANOVA with Tukey's adjustment for multiple comparisons.

Next, we determined the binding profile of the chimeric toxins for Gb3. Previous reports showed that Stx1a binds Gb3 in a dose-dependent manner and with a higher affinity than Stx2a [28], [46]. When the chimeric toxins were examined for the capacity to bind Gb3 by ELISA, we found that the Gb3-binding capacity of the chimeric toxins was again dependent on the origin of the B-subunit. Stx1a and 211 bound Gb3 with a significantly greater affinity than Stx2a or 122 (p<0.01) (Figure 2).

**Figure 3:**
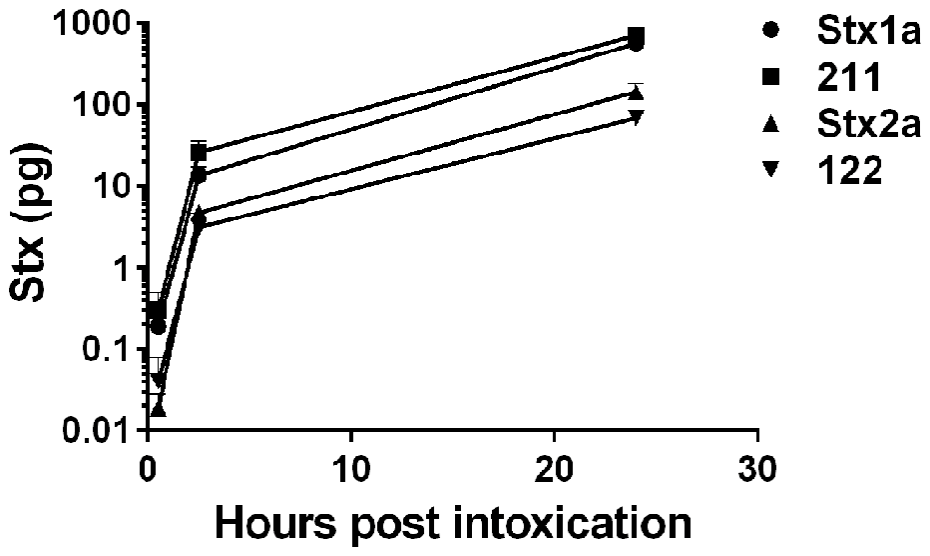
Stx translocation through a polarized HCT-8 cell monolayer after 20 ng was applied to the apical chamber. The Stx1a B subunit was associated with statistically greater toxin translocation compared to the Stx2a B subunit. A greater amount of Stx1a and 211 was recovered from the basolateral chamber as compared to Stx2a and 122 at 2.5 and 24(p<0.05). Error bars represent SEM. n = 4 biological replicates

Our next step was to investigate the translocation properties of the toxins through polarized HCT-8 cell monolayers by the procedure published by Hurley *et al*. [45]. As expected, the HCT-8 cells remained polarized for 24 hours after 20 ng of any of the Stxs were applied to the apical transwell chamber (data not shown). However, a significantly greater amount of Stx1a or 211 translocated through the HCT-8 polarized monolayers to the basolateral chamber than did Stx2a or 122 at 0.5, 2.5, and 24 hours post intoxication (p≤0.05) (Figure 3). For example, after 24 hours, 0.57 and 0.71 ng of Stx1a and 211, respectively, had translocated while only 0.14 and 0.07 ng of Stx2a and 122, respectively, were recovered from the basolateral chamber.

### Ip lethality of the toxin panel

We next determined the ip LD_50_s of the toxin panel. The ip LD_50_ values for Stx1a and Stx2a were 430 ng and 2.2 ng respectively (Table 1), values that are consistent with previous reports [26]. The 122 chimeric had an equivalent LD_50_ to that of Stx2a at 2.3 ng (p>0.05), while 211 had an LD_50_ closer to that of Stx1a at 2300 ng, results that again show that the relative toxicity of the chimeric is dependent on the source of the B subunit. The LD_50_s corresponding to the Stx2 B subunit were statistically lower compared to LD_50_s associated with the Stx1 B subunit (p<0.05). The mean time-to-death (MTD) for ip intoxicated mice was analogous for all four toxins, at approximately 4.3 days.

**Table 1:** Ip LD_50_ of native and chimeric toxins.

Toxin	LD_50_, ng*	95% CI*
Stx1a	430	250–620
211	2300	1300–4600
Stx2a	2.2	2.0–2.6
122	2.3	1.7–3.1

*LD_50_ and confidence intervals were determined by Probit analysis after taking the log of the values.

### Oral intoxication of chimeric Stxs

**Figure 4:**
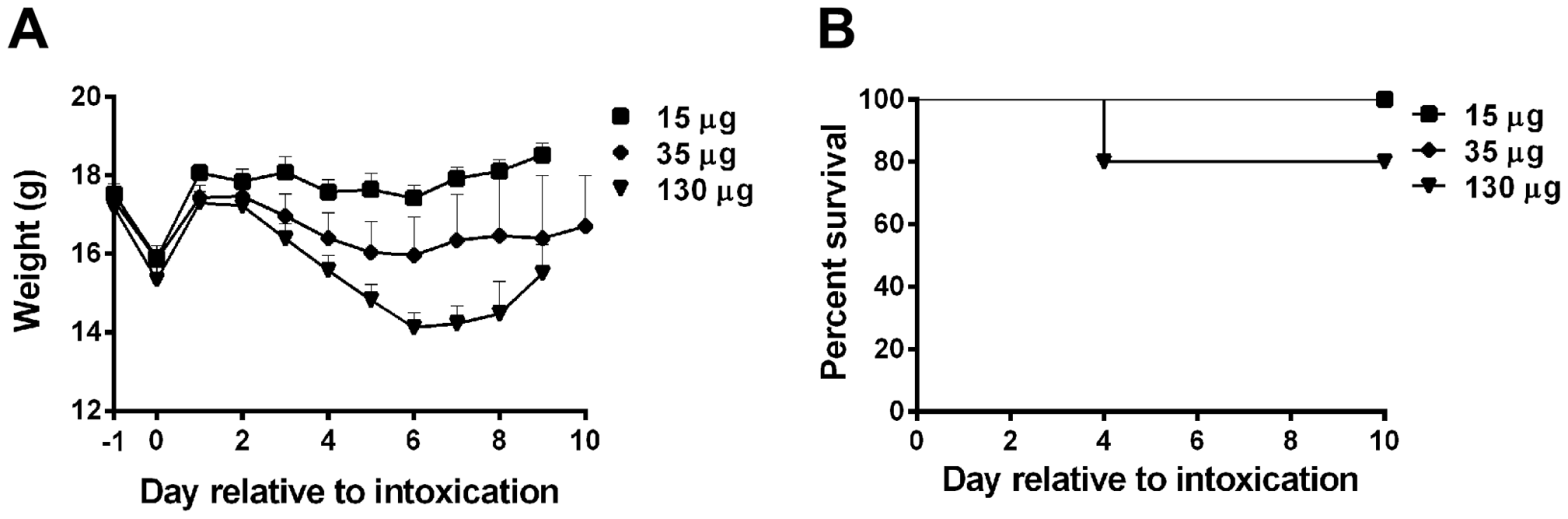
Morbidity and mortality after ig intoxication with chimeric 122. Ig intoxication of up to 15 μg 122, 5X the Stx2a LD_50_ of 2.9 μg [40], did not result in morbidity or mortality. Weight loss was observed after intoxication with 35 and 130 μg 122 (A) and one animal in the 130 μg group succumbed to intoxication on day 4 (B). Error bars represent SEM; 15 μg: n = 10; 35 and 130 μg: n = 5

We continued our in vivo analysis of the chimeric Stxs with a determination of the oral toxicity of those preparations. We recently reported that the i.g. LD_50_ of Stx2a is 2.9 μg/mouse [40]. Although extreme weight loss and death were observed at Stx2a concentrations of 2 μg or greater, we observed only minimal weight loss and no mortality with 122 at doses of 15 μg or below (data not shown). We next gavaged 35 or 130 μg of 122 into new groups of mice (approximately 10- or 40-times the Stx2a ig LD_50_, respectively). At the 35 and 130 μg doses of 122, we observed weight loss in both groups and one death in the 130 μg cohort (Figure 4A & B). From these results we concluded that the 122 ig LD_50_ is greater than 130 μg and more than 10-times the Stx2a ig LD_50_. We found no morbidity or mortality after intragastric administration of 157 μg Stx1a [40] or 211 at doses up to 70 μg (data not shown).

### Higher percent of active Stx2a recovered than active Stx1a from feces

**Figure 5:**
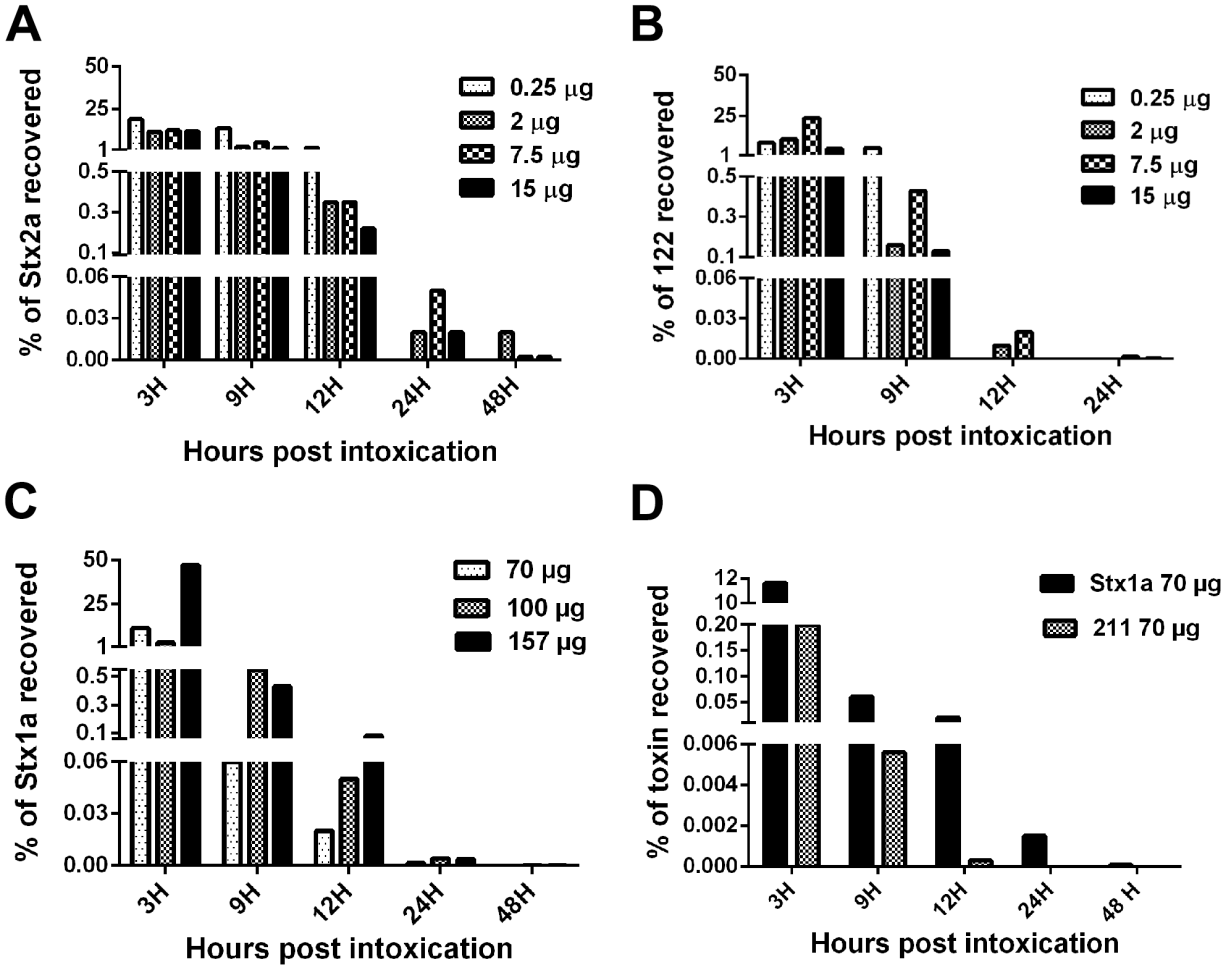
The percent of active toxin recovered from feces compared to toxin input. The amount of active toxin detected was divided by the amount administered and multiplied by 100 to determine the percent of active Stx2a (**A**), 122 (**B**), Stx1a (**C**), or 211 (**D**) recovered from the stool. A significantly greater amount of active Stx2a was recovered than active 122 at 9, 12, 24 hours post intoxication at all concentrations fed (t-Test p<0.05). A significantly greater amount of active Stx1a was recovered than active 211 at three, nine, and 12 hours post intoxication (Students -Test p≤0.05).

To determine whether the toxins were shed differentially into the feces, or if large amounts of the toxins were shed into the stool after oral intoxication, we collected stool from mice fed Stx1a, Stx2a, 211, or 122 at 3, 9, 12, 24, and 48 hours post intoxication and measured the cytotoxicity from those samples on Vero cells. Fecal samples were also collected from control mice to determine the baseline toxicity of stool for Vero cells. After subtracting the background fecal toxicity, we determined the CD_50_/mL attributable to the active toxins in stool. Once we measured the CD_50_ for each toxin present in the stool, we used the specific activity of the toxins to calculate the actual amount of toxin (μg) excreted at each time point. The highest levels of toxin in stools were found at the three hour time point for all four toxins. Active Stx2a was recovered from mice gavaged with 2, 7.5, or 15 μg of Stx2a for up to 48 hours post intoxication, whereas the toxin was only detectable in the feces of mice given 0.25 μg ig for 12 hours (Figure 5A). Chimeric 122 was only detected up to 24 hours post intoxication, and there was a significant reduction in toxin excreted from three to nine hours (p<0.05) (Figure 5B). Stx1a was also detectable in the feces up to 48 hours post-intoxication (Figure 5C), while chimeric 211 was only found in the first 12 hours (Figure 5D). When the calculated toxin output was compared to the initial intoxication dose, we found that a relatively low percentage of each Stx was recovered from stool. For example, when 15 μg of Stx2a were given ig, we detected 1.8 μg in the feces at three hours post-intoxication. Levels of active Stx2a in fecal samples were recovered at a higher percentage of the initial dose over time compared to the rest of the toxin panel (Figure 5A–D), except for the 157 μg Stx1a dose at the three h timepoint. Overall, we recovered a higher percentage of active native Stxs compared to the chimeric Stxs when equivalent amounts of toxin were gavaged (Figure 5A–D). In particular, significantly less 122 than Stx2a was detected at three hours in the 0.25 and 15 μg groups and for all groups at the remaining time points (p<0.05) (Figure 5A &B). In addition, significantly more Stx1a than 211 was recovered from the groups that received 70 μg at all time points (p<0.05) (Figure 5D). We next tried to determine the total concentration of toxin in the feces (active and possibly inactive) with an Stx ELISA. However, the levels of Stx in the feces were below the high limit of detection in the ELISA (not shown) [47].

### Chimeric toxin stability was reduced in acidic conditions

Because we observed that the lethality of the chimeric toxins was reduced *in vivo* as compared to the native toxins, especially after ig intoxication, and since previous reports indicated that holotoxin stability is critical for toxin activity [36], we tested the stability of the toxin panel after exposure to acidic conditions. We found that while none of the toxins exhibited altered toxicity when incubated at pH 7 or pH 5 as compared to PBS control (data not shown), the entire toxin panel showed reduced toxicity when incubated in pH 3 buffer at 37°C or 60°C, with a greater reduction in activity at 60°C. The chimeric toxins exhibited an increased susceptibility to inactivation at pH 3 when compared to the native toxins (Table 2). At both 37°C and 60°C, the reduction in activity of chimeric Stxs was significantly greater than the reduction observed in the native Stx with a corresponding B subunit (p<0.05).

**Table 2:** Fold change of Vero cytotoxicity after incubation in a buffer of pH(PBS) for 1 h at 37 C or 60 C.

Stx type	37°C	60°C
Stx1a	1.0^a^	0.79^a^
211	0.88^a^	0.46^a^
Stx2a	0.94^b^	0.62^b^
122	0.74^b^	0.48^b^

a,b The reduction in activity of the chimeric was significant at both temperatures (P<0.05).

## Discussion

The major finding from this study was that the toxicity difference between Stx1a and Stx2a on cells and ip in mice is mediated by the B subunit. For this investigation, we evaluated purified chimeric toxins in which both the A_2_ peptide and B subunit originated from the same native toxin. Although another group had created chimeric Stxs with hybrid A_1_/A_2_ subunits, they used plasmid complementation to combine the chimeric A_1_/A_2_ with the B subunit, and the cytotoxic activities of those chimerics were significantly lower than those of the native Stx with the corresponding B subunit [48]. The structural design of our chimeric toxins is likely the reason that they exhibited *in vitro* cytotoxicity equivalent to the native toxin with the corresponding B subunit. We also believe it was critical that we quantitated the toxin level in purified preparations based on toxin antigen rather than total protein concentration. Other groups of investigators who examined Stx1a/Stx2a chimeras either reconstituted the toxins from purified subunits and observed lower cytotoxicity values than expected [25] or did not get the expected difference in toxicity between Stx1a and Stx2a [33], or they used an operon fusion method but did not produce an active Stx1aA/Stx2aB hybrid [34], [35], or they focused solely on the capacity of Stx2a and the Stx1aA/Stx2aB chimeric to bind human serum amyloid component P (HuSAP) [49].

Although our finding that the B subunit is responsible for the differential toxicity of Stx1a and Stx2a on Vero and HCT8 cells and ip in mice was not surprising based on previous studies [27], [33]–[35], our investigation was the first in which a complete set of toxins and chimeras (Stx1a, Stx2a, and each hybrid, 211 and 122) were compared in both *in vitro* and *in vivo* assays. Furthermore, this is the first report in which the Vero cell specific activity of the chimeric toxins was equivalent to the activities associated with the native toxin. However, we found that although Stx2a and 122 have an identical mouse LD_50_ by the ip route, the 211 chimeric demonstrated about a 5-fold higher ip LD_50_ than did Stx1a. In contrast, Shimizu *et al*. found that the LD_50_ of an Stx2aA/Stx1aB hybrid is approximately 2-fold lower than that of Stx1a [35], perhaps because that hybrid bound to Gb3 with a lower affinity in their hands than did Stx1a, and a lower binding affinity to Gb3 is associated with a lower LD_50_ in mice [32]. The decreased *in vivo* lethality as compared to *in vitro* toxicity of 211 in our hands may be due to a reduced stability of the holotoxin as compared to Stx1a as measured by sensitivity to pH, and perhaps exacerbated *in vivo* by the requirement of the toxin to travel from the peritoneal cavity to the kidney. By comparison, *in vitro* the toxin is overlaid directly onto the target cell and is not subject to multiple potentially harsh environments.

We previously established that the oral mouse intoxication LD_50_ for Stx2a is 2.9 μg, 1,000 times greater than the ip LD_50_ [40]. However, in this study we found that 122 was not lethal to mice at 35 μg. Nevertheless, mice gavaged with 130 μg of 122 displayed morbidity profiles similar to mice gavaged with 2 μg of Stx2a and one mouse died. These observations indicate that 130 μg of 122 is close to the ig LD_50_. We anticipate that the ig LD_50_ of 211 would be at least 1,000 times greater than the ip LD_50_ (as we found for Stx2a),and, therefore, more than 2.3 mg/mouse.

We hypothesize that oral intoxication is likely the most strenuous test of the activity of the toxins because the molecule must pass through the gastrointestinal tract (GI) before entering the bloodstream. As a corollary to that theory, we initially speculated that 1,000 times more toxin is required for ig compared to ip intoxication because a portion of toxin is inactivated by the acidic conditions in the intestines. We found however, that less than 25% of the Stx2a and 50% of Stx1a gavaged, respectively, could be found in the stool, a result that indicates that most of the active Stx is not combined into stool. Overall we recovered a greater percentage of active Stx2a than Stx1a from stool, even though a higher amount of Stx1a was gavaged and Stx2a was more lethal than Stx1a. These latter differences between the native toxins may be due to unique binding and/or translocation patterns during transit through the GI tract, and we did observe greater Stx1a binding to Gb3 and translocation in the HCT-8 cells *in vitro*. We do not believe a significant reduction in activity of the native Stxs occurred in the GI tract, as we did not find Stx antigen in the stool at levels above that detected by Vero cell assay, and because we and others showed that the toxins are stable at pH 3 and 37°C [50]. Additionally, the Stxs were not readily inactivated by proteases in the mucus *in vitro* (data not shown). We believe, therefore, that after oral intoxication, a combination of factors results in less toxin reaching the target cells in the kidney, which therefore results in an increased ig LD_50_ compared to the ip LD_50_.

Our results indicate that the B subunit of each toxin is critical for the differential toxicity between Stx1a and Stx2a and, further, that the B subunit is particularly critical for proper delivery of the toxins from the GI tract. However, a major question still remains: why is Stx2a more potent in vivo that Stx1? Our findings suggest that the differential mouse lethality of Stx1a and Stx2a may occur at the level of dissemination from the GI tract within the animal or directly at the level of toxicity to the kidney. That Stx2a has a 1000-fold lower CD_50_ for renal endothelial cells than Stx1a suggests the difference occurs at the site of the kidney [51].

## Supporting Information

Figure S1 **Stx subunits are stained in a linear manner by Oriole fluorescent stain.** (A) Oriole stained SDS-Page gel of Stx1a (lanes 1–3) and Stx2a (lanes 4–6) run at equal concentrations as follows: lanes 1 and 4: 13 μg; lanes 2 and 5: 6.5 μg; lanes 3 and 6: 3.25 μg. The full A subunit is approximately 35 kDa and the cleaved A_1_ subunit is approximately 32 kDa. The B subunit is approximately 7 kDa. The two-fold dilutions demonstrate a similar dose dependent fluorescent staining intensity for both Stxs. (B) Linearity of the Oriole stain. Stx concentration graphed against fluorescent intensity (arbitrary units).
(TIF)
