# Supplementary figures and images for: Comparisons of Native Shiga Toxins (Stxs) Type 1 and 2 with Chimeric Toxins Indicate that the Source of the Binding Subunit Dictates Degree of Toxicity
Source: PLoS One. 2014 Mar 26;9(3):e93463. doi: 10.1371/journal.pone.0093463 (PMC3966898; doi:10.1371/journal.pone.0093463)

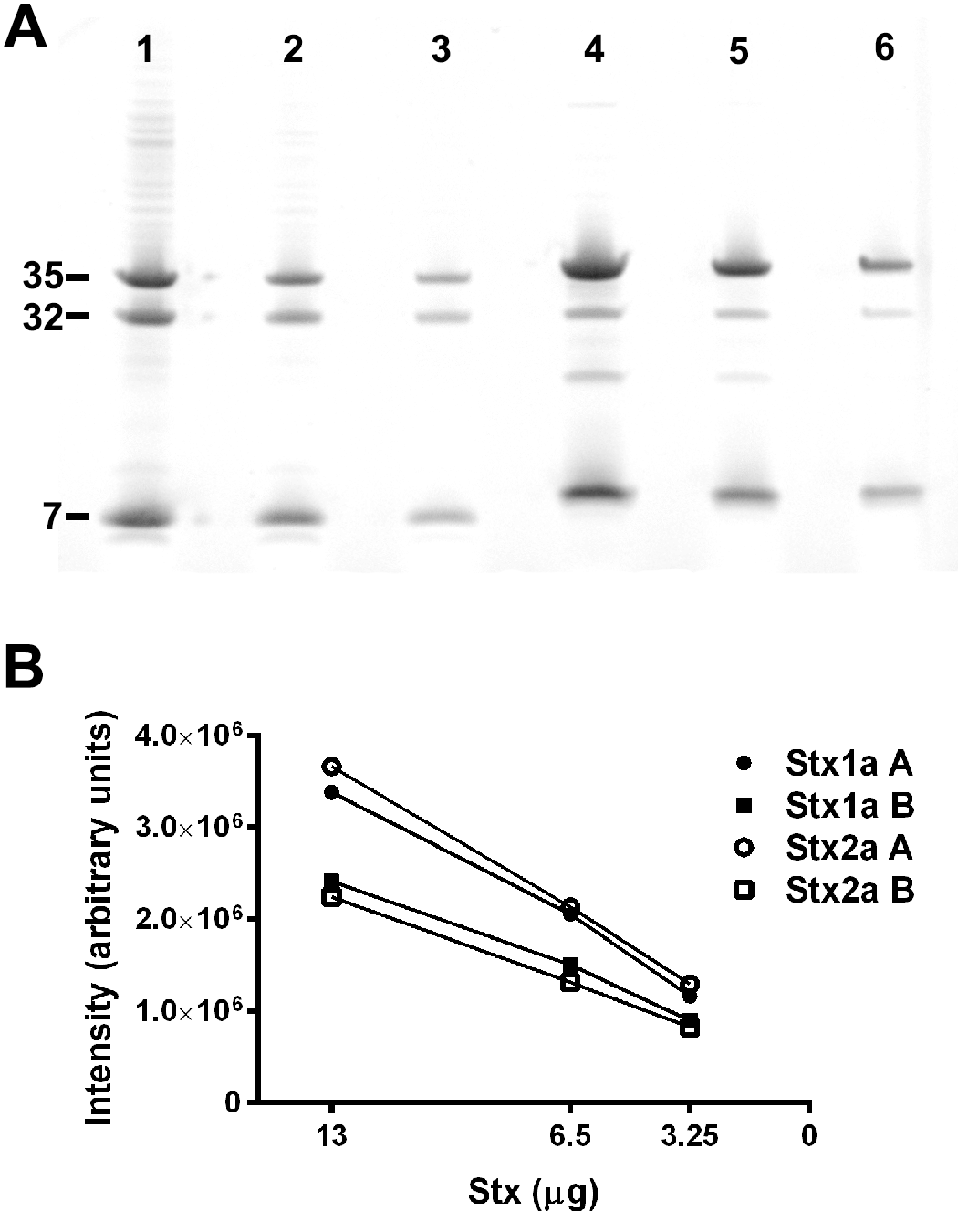

Supplement: Figure S1 — Stx subunits are stained in a linear manner by Oriole fluorescent stain. (A) Oriole stained SDS-Page gel of Stx1a (lanes 1–3) and Stx2a (lanes 4–6) run at equal concentrations as follows: lanes 1 and 4: 13 μg; lanes 2 and 5: 6.5 μg; lanes 3 and 6: 3.25 μg. The full A subunit is approximately 35 kDa and the cleaved A1 subunit is approximately 32 kDa. The B subunit is approximately 7 kDa. The two-fold dilutions demonstrate a similar dose dependent fluorescent staining intensity for both Stxs. (B) Linearity of the Oriole stain. Stx concentration graphed against fluorescent intensity (arbitrary units). (TIF) [file pone.0093463.s001.tif]
